# Etiological myocardial injury classification versus binary myocardial infarction classification in the multi-ethnic study of atherosclerosis (MESA)

**DOI:** 10.1016/j.ajpc.2026.101525

**Published:** 2026-03-04

**Authors:** Shubham Tomar, Karita C.F. Lidani, Matthew Dekker, Aline A.I. Moraes, Wendy S. Post, Patrick J. Trainor, Craig W. Johnson, Susan R. Heckbert, Roger S. Blumenthal, Robyn L. McClelland, Yunbi Nam, Khurram Nasir, Michael J. Blaha, Andrew P. DeFilippis

**Affiliations:** a Vanderbilt University Medical Center, Nashville, TN, USA; b University of Washington, Seattle, WA, USA; c Jefferson Einstein Hospital, Philadelphia, PA, USA; d Johns Hopkins Ciccarone Center for the Prevention of Cardiovascular Disease, Baltimore, MD, USA; e Virginia Commonwealth University, Richmond, VA, USA; f Methodist DeBakey Heart & Vascular Center, Houston, TX, USA

**Keywords:** Myocardial injury, Myocardial infarction, Adjudication, Fourth universal definition of myocardial infarction, Type 2 myocardial infarction, Acute non-ischemic myocardial injury

## Abstract

**Background:**

Prospective cohorts almost exclusively classified acute myocardial injury as a binary entity—presence or absence of myocardial infarction (MI).

**Objective:**

To compare Binary Classification of MI used in the Multi-Ethnic Study of Atherosclerosis (MESA) to an Etiological Classification of myocardial injury.

**Methods:**

Comparison of two different MI adjudication systems in 6809 participants aged 45–84 years old from MESA, a community-dwelling cohort free of cardiovascular disease at enrollment (2000). The Binary Classification defined events as MI versus "No MI". The Etiological Classification, based on the Fourth Universal Definition of MI, categorized events as MI (types 1–5), acute non-ischemic myocardial injury, chronic myocardial injury, and no myocardial injury. Number and distribution of myocardial infarction/injury events identified by each classification system were compared.

**Results:**

Binary Classification identified 333 MI events, while Etiological Classification identified 406 MIs, of which 202 (49.8%) were type 1 MI and 182 (44.8%) were type 2 MI, 22 (5.4%) were other MI types. Notably, 147 events (33 type 1 MI, 105 type 2 MI, 9 other MI types) were newly captured as MI with the Etiological Classification that were classified as No-MI by the Binary Classification. Among the 333 MI identified by the Binary Classification, 51% were type 1 MI (*N* = 169), 23% as type 2 MI (*N* = 77) and 17% as acute non-ischemic myocardial injury (*N* = 55) in the Etiological Classification.

**Conclusions:**

Etiological Classification identifies additional, etiologically distinct myocardial injury events. Almost half of the events identified as an MI by Binary Classification were identified as type 2 MI or acute non-ischemic myocardial injury by Etiological Classification.

## Highlights

• Epidemiology of myocardial injury events beyond myocardial infarction (MI) as a binary entity is lacking.
• The Universal Definition of MI classifies a spectrum of myocardial injury events based on etiology.
• MI is classified by etiology in clinical practice.
• Etiological Classification will enable research across the full spectrum of myocardial injury and will allow for the discovery of myocardial injury event type specific precedent risk factors.

**Figure:**
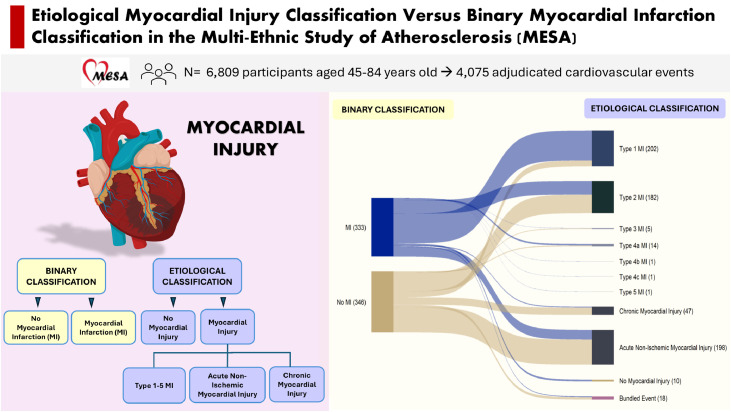
Central Illustration.

## 1 Introduction

Each year, over 12 million patients present to emergency departments in North America and Europe with suspected acute myocardial infarction (MI) [1]. The reported incidence rate of MI can vary significantly based on the criteria used to define an MI [2]. Since the first global definition of MI given by the World Health Organization (1950–1970) [3], MI has largely been classified as present or absent. However, the understanding of MI has evolved, and it has been recognized for decades that MI is a heterogeneous group of etiologically distinct entities.

The 4^th^ Universal Definition of MI (4^th^ UDMI) defines myocardial injury as an umbrella term, identified by an elevation in cardiac troponin (cTn) [3]. Myocardial injury can be acute (with dynamic cTn changes) or chronic (stable cTn elevations). Acute myocardial injury is further classified as an MI when caused by ischemia or as acute non-ischemic myocardial injury in the absence of ischemia. MI is further classified based on etiology into atherothrombotic plaque disruption (Type 1 MI), non-atherothrombotic myocardial oxygen supply demand mismatch (Type 2 MI), sudden cardiac death related (Type 3 MI), percutaneous coronary procedure related (Type 4a-c MI), or cardiac surgery related (Type 5 MI) [[3], [4], [5]].

Despite differing prevalence, pathobiology, and prognosis, most prospective observational cohort studies adjudicate acute MI as a singular entity, as opposed to etiologically distinct MI types. Major studies like the International Study of Infarct Survival (ISIS) [6], the Framingham Heart Study (FHS) [7], and the Systolic Blood Pressure Intervention Trial (SPRINT) [8] have reported outcomes based on the presence or absence of MI, raising concerns about the generalizability of their findings across all myocardial injury types as defined by the 4^th^ UDMI. Utilizing an etiology-based classification system in a community-based cohort will allow for a seminal understanding of the incidence of distinct myocardial injury types and associated predisposing risk factors in the general population. A cohort study of people living with Human immunodeficiency virus (HIV) illustrates the value of this approach, demonstrating that the incident type 2 MI accounts for nearly half of all MIs in this specific patient population [9].

In the absence of adjudication of the full spectrum of myocardial injury events in cardiovascular research, the relevance of risk factors, diagnosis, treatment and prevention strategies associated with etiologically specific myocardial injury event types (i.e. type 1 versus type 2 MI) remain unexplored [2]. To fill this knowledge gap, we performed standardized physician re-adjudication of all suspected clinical cardiovascular events in the Multi-Ethnic Study of Atherosclerosis (MESA) as MI types 1–5, acute non-ischemic myocardial injury, and chronic myocardial injury. We present an analysis comparing this etiology-based classification (herein called “Etiological Classification”) to the existing MESA MI adjudication system (since 1999), which classifies MI as a single entity (Yes/No), (herein called “Binary Classification”). We hypothesize that MI events in Binary Classification would result in a heterogeneous group of myocardial injury events as per the Etiological Classification.

## 2 Methods

### 2.1 Study population

MESA is a large, ongoing, community-based prospective cohort study funded by the National Heart, Lung, and Blood Institute (NHLBI) and designed to investigate the prevalence, causes, and progression of subclinical and clinical cardiovascular disease (CVD) in the United States. Between 2000 and 2002, MESA enrolled 6809 participants aged 45 to 84 years, free of clinically apparent CVD at baseline. The cohort includes an equal number of men and women from four self-described major racial/ ethnic groups—White, Black, Hispanic, and Chinese— recruited from six U.S. communities [[10], [11]].

### 2.2 Event selection

MESA identifies potential cardiovascular events through a combination of participant self-reports during in-person examinations or clinic visits, interim follow-up telephone interviews conducted every 9 to 12 months, and direct notifications from participants to field centers following an event. For participants who were lost to follow-up, the Coordinating Center monitors obituaries or public records to identify deaths. MESA identifies and reviews all clinical events to determine their need for comprehensive evaluation by a physician adjudication committee. Each event is assessed using clinical documentation such as physician notes, hospital records (e.g., discharge summaries, lab results including cardiac biomarkers), and billing codes to establish a list of all ICD- 9/10 codes associated with the event independently or with help from a nosologist. Events are selected for physician adjudication either by a pre-defined computer algorithm that identifies relevant International Classification of Disease (ICD) 9/10 codes or by field center specialists who identify potential cardiovascular involvement, even in the absence of qualifying diagnostic codes. This selection process is designed to maximize sensitivity to capture all potential cardiovascular events [[12], [13]]. Additional details about this process can be found in the supplement.

In addition to the collection of event source documents, specific cardiovascular data are systematically sought, abstracted, and organized into case packets for physician adjudication. This includes data on presenting symptoms, ECGs, cardiac biomarkers (e.g., cardiac troponins, creatine kinase-myocardial band (CK-MB), total creatine kinase (CK)), coronary angiography, and assay reference ranges [12].

### 2.3 Binary classification

The Binary Classification system has been in use since the start of the MESA in 1999. Criteria for classification include a combination of clinical symptoms (i.e., chest pain), biomarker levels (CK-MB, total CK, lactate dehydrogenase, and troponins) and ECG findings (**Supplemental Tables 4 and 5**). Events are classified as “MI” (Definite or Probable MI), or "No MI” based on standardized criteria. Adjudication is performed by two physician reviewers; with disagreements resolved by discussion between the two reviewers or, for rare unresolved cases, by a third reviewer. Additional details are provided in the supplement.

### 2.4 Etiological classification

All events meeting criteria for a possible cardiovascular event necessitating physician adjudication per MESA protocol during the first 14 years of follow-up (15^th^ July 2000 through 31^st^ December 2014) were adjudicated with the Etiological Classification system as a part of a study funded by the National Institutes of Health (R01HL158976) [14]. The Etiological Classification was based on taxonomy defined by the 4^th^ UDMI. The adjudication process involved systematically abstracting existing event-specific data from the time of event, including detailed information on symptoms, ECG, cardiac biomarkers (cTn, CK), coronary angiography and event-specific cardiac biomarker assay reference ranges. This adjudication was performed between 2022 and 2025 and was blinded and independent of any MESA study data, including previous MI adjudication.

Acute myocardial injury was defined as a relative change of at least 20 % in cTn concentrations from baseline, in cases where baseline cTn was elevated, or at greater than 50 % change when cTn concentration was below the reported upper limit of normal. In approximately 7 % of adjudicated events cTn was not available, and total CK or CK-MB values were used. MESA began before the 99^th^ percentile URL was widely adopted as the standard for abnormal cardiac troponin. Adjudicators were limited to on-site-reported assay decision limits for cTn, which may differ from the manufacturer's 99^th^ percentile URL. The Etiological Classification system instructed adjudicators to use their best judgment when interpreting troponin value ranges not consistent with current standards (99^th^ percentile of a reference population). Acute myocardial injury caused by myocardial ischemia was categorized as MI. The presence of ischemic symptoms could aid in determining ischemia but was not considered definitive and was particularly difficult to assess among individuals who were sedated, obtunded, or in the perioperative state. ECG and echocardiography provided supportive evidence when available. Evidence of a condition capable of causing significant ischemia via myocardial oxygen supply/demand mismatch (e.g., sustained tachycardia, hypoxia, hypotension, severe anemia, coronary spasm), was also considered in the diagnosis of type 2 MI. In the absence of clear evidence of ischemia as the cause of an acute myocardial injury, we favored assigning a diagnosis of acute nonischemic myocardial injury [12]. Additional details of the Etiological Classification, including criteria for defining other types of myocardial injury have been previously published [12] and are described in **Supplemental Table 1.**

Each event was independently reviewed by at least two cardiologists [events randomly distributed and assigned amongst A.P.D, M.J.B, K.N]. Classification required agreement between two blinded cardiologists using a standardized web-based interactive myocardial injury adjudication tool specifically designed for myocardial injury identification and type adjudication [12]. If there was discordance between the initial two adjudicators, the event was assigned to a third cardiologist. If all three cardiologists disagreed, the event was independently reviewed by an executive committee, which included a lead author of the 4^th^ UDMI, a peer reviewer for the 4^th^ UDMI, and a senior myocardial injury researcher. In these instances, consensus on event diagnosis was reached through discussions among all three executive committee members (**Supplement Fig. S1**)[12]. At least one of the following were available for all suspected myocardial injury events: an ECG, cardiac imaging, or a cardiac biomarker; and 94 % of suspected events had both ECG and cardiac biomarkers (**Supplement Fig. S2, Supplement Table 2**) [12].

### 2.5 Bundle events

In the Binary Classification, if a potential link between two events were noted by MESA Surveillance and Events staff, the events were flagged and noted for combined assessment. In contrast, in the Etiological Classification, all events were independently adjudicated first, and then all events occurring within 28 days of discharge from a prior hospitalization were identified and re-reviewed together. These event pairs were evaluated together by two cardiologists independently to determine whether the subsequent event represented a continuation of the previous episode or a distinct, independent event. In cases of disagreement, a third cardiologist reviewed the information from both events to reach a consensus. If the subsequent event was deemed a continuation, it was designated as a "Bundled Event," and the first event retained the original diagnosis.

### 2.6 Statistical analysis

Descriptive statistics are presented using tables and Sankey plots to illustrate the distribution of events under both the Binary Classification and Etiological Classifications. For all analyses, no distinction was made between a definite or probable MI, both were considered as MI in the Binary Classification system. All statistical analyses were conducted using R software, version 4.4.3 (2025).

## 3 Results

**Fig. 1:**
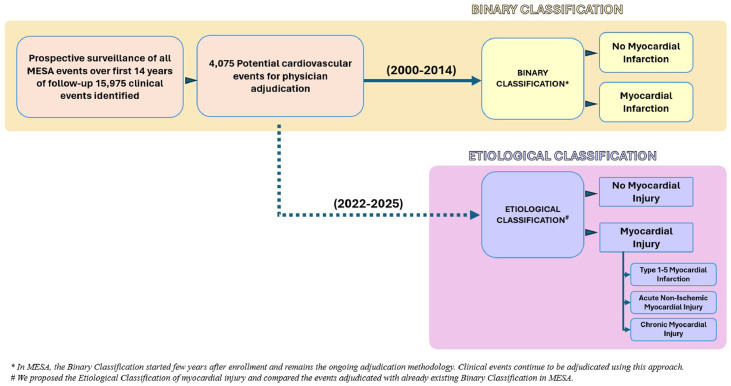
Event selection for different physician adjudication methodologies in the Multi-Ethnic Study of Atherosclerosis.

**Fig. 2:**
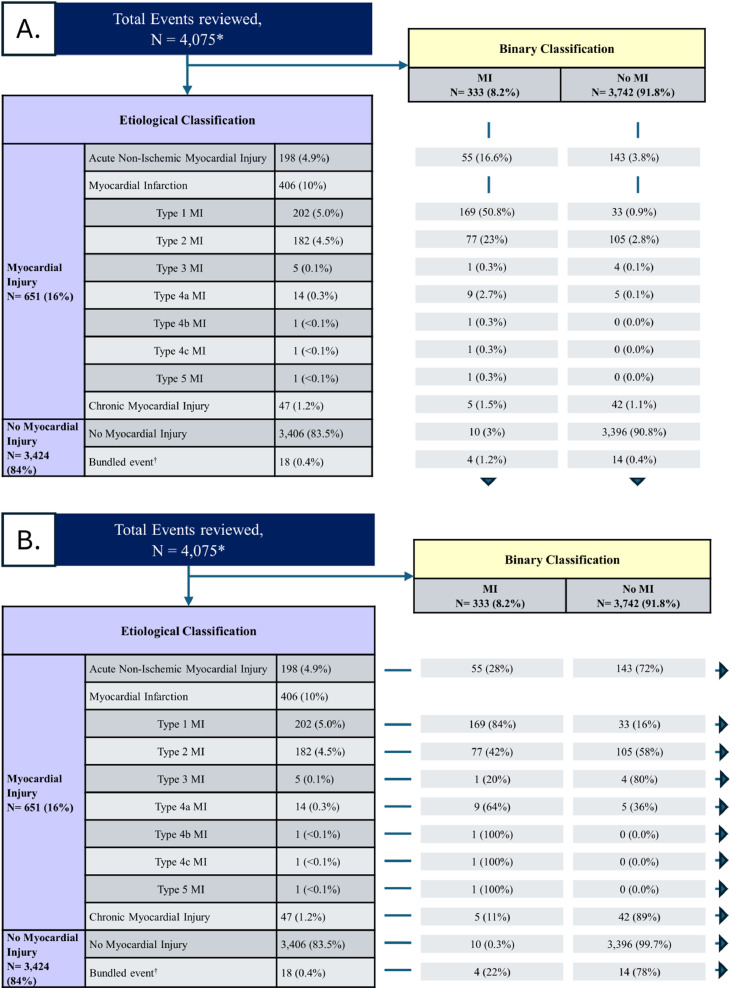
Etiological Classification and Binary Classification of clinical events in the Multi-Ethnic Study of Atherosclerosis (MESA) (A. Column percentages and B. Row percentages).

**Fig. 3:**
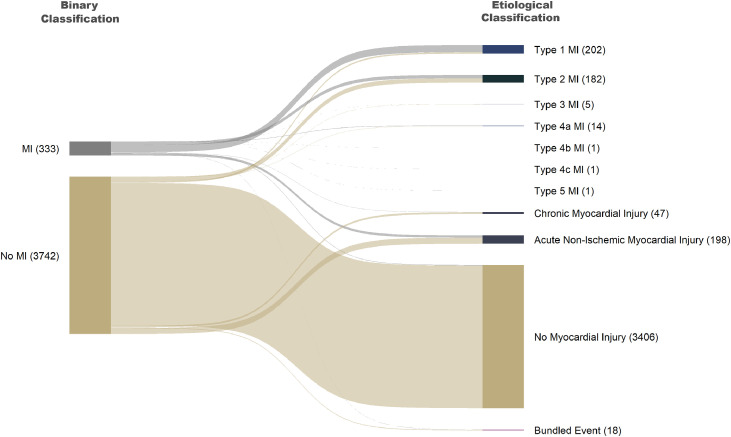
Sankey plot showing the distribution of cases adjudicated as a myocardial injury event by the Etiological Classification system. Binary Classification of MI on left (with combined Definite and Probable class) and Etiological Classification of myocardial injury on right.

**Fig. 4:**
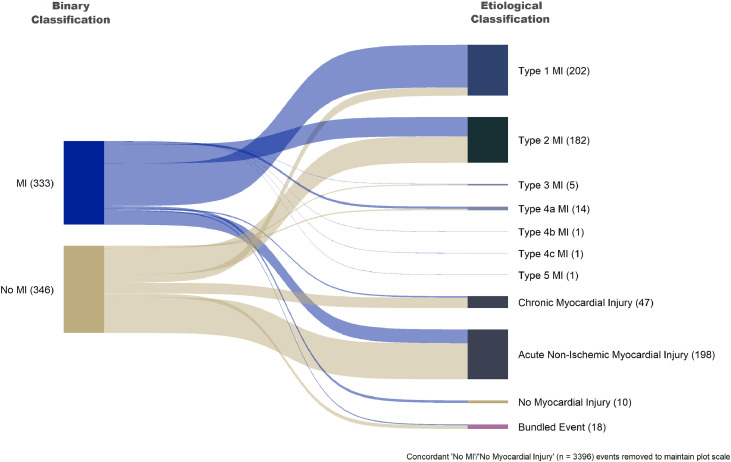
Sankey plot showing the distribution of cases adjudicated as a myocardial injury event by the Etiological Classification system. Binary Classification of MI on left and Etiological Classification of myocardial injury on right.

Note: All concordant No MI/ No Injury events (*N* = 3396) are removed. Multi-Ethnic Study of Atherosclerosis (MESA); Myocardial Infarction (MI).

The MESA includes 6809 participants with a mean age of 62.2 ± 10.2 years, including 53 % women. The cohort represents four self-identified major racial/ethnic groups: White (39 %), Black (28 %), Hispanic (22 %), and Chinese (12 %). Between 2000 and 2014, MESA recorded and reviewed 15,905 clinical events, of which, 4075 were identified as potential cardiovascular events that met the criteria for physician adjudication (Fig. 1). Among these 4075 events, 333 (8.2 %) were previously adjudicated as an MI by the Binary Classification (Fig. 2). Using the Etiological Classification, myocardial injury was identified in 651 (15.9 %) of the 4075 events, including 406 MI and 198 acute non-ischemic myocardial injury events (Fig. 2, Fig. 3, Fig. 4). Chronic myocardial injury was present in 47 events. Among the 406 MI identified with the Etiological Classification, 202 (50 %) were type 1 MI, 182 (45 %) were type 2 MI, and 22 (5 %) were other MI types (Fig. 2).

*Total 15,905 clinical events between 2000–2014 in MESA. Among these, 4075 potential cardiovascular events that met the MESA criteria for physician adjudication. Column Percentages: Each cell shows the % of events within the Binary Classification category that fall into the corresponding Etiological Classification category. Row Percentages: Each cell shows the % of events within the Etiological Classification category that fall into the corresponding Binary Classification category. ^†^ Bundled events are recurrences of myocardial injury event occurring within 28 days of discharge from a previous event. For analysis, these are considered a continuation of previous myocardial injury events and are classified as no myocardial injury. Myocardial Infarction (MI); Multi-Ethnic Study of Atherosclerosis (MESA).

Multi-Ethnic Study of Atherosclerosis (MESA); Myocardial Infarction (MI); Universal Definition of Myocardial Infarction (UDMI).

Among the 604 acute myocardial injury events (acute non-ischemic myocardial injury, MI type 1–5) identified using the Etiological Classification, 482 (79.8 %) were first events, while 122 (20.2 %) were subsequent events. Eighteen (3 %) acute myocardial injury events were adjudicated as bundled events in the Etiological Classification (Fig. 2 **and Supplemental Table 6**).

The Binary Classification initially identified 333 MI events, amongst which 51 % were adjudicated as type 1 MI (*N* = 169), 23 % as type 2 MI (*N* = 77) and 17 % as acute non-ischemic myocardial injury (*N* = 55) using the Etiological Classification (Fig. 2**A**). Among 3742 No MI events based on Binary Classification; 290 (7.7 %) events were classified as an acute myocardial injury: *N* = 143 (49 %) acute non-ischemic myocardial injury, *N* = 105 (36 %) type 2 MI, *N* = 33 (11 %) type 1 MI, and *N* = 9 (3 %) other MI subtypes (Fig. 2**A**). From the Binary Classification, 10 MI were adjudicated as no myocardial injury events in the Etiological Classification. Four MI events from the Binary Classification were re-adjudicated as bundled events in the Etiological Classification (Fig. 2, Fig. 3, Fig. 4).

Among the 202 type 1 MIs identified by the Etiological Classification, 84 % (*N* = 169) were identified as an MI by the Binary Classification (Fig. 2**B**). Among the 182 types 2 MIs identified by the Etiological Classification, 42 % (*N* = 77) were identified as an MI by the Binary Classification (Fig. 2**B**). Of the 198 acute non-ischemic myocardial injury events identified by the Etiological Classification, 28 % (*N* = 55) were identified as an MI by the Binary Classification (Fig. 2**B**). Among 3406 unbundled No Myocardial Injury events based on Etiological Classification, 10 (0.3 %) were adjudicated as MI by the Binary Classification (Fig. 2**B**).

## 4 Discussion

In a large prospective cohort of community-dwelling adults, the Etiological Classification of myocardial injury (2022–2025), based on the taxonomy of the 4^th^ UDMI, resulted in capturing and classifying a broader and heterogeneous spectrum of etiologically distinct acute myocardial injury events as compared to a Binary Classification of MI (2000–2014). Approximately fifty percent of MIs captured with the Binary Classification were classified as type 1 MI by the Etiological Classification system, with type 2 MI and acute non-ischemic myocardial injury constituting the other half. By creating “clean” phenotypes, the Etiological Classification of myocardial injury utilized in this study will advance the understanding of the epidemiology of all acute myocardial injuries, including all five types of MI, in the US communities.

In a prior study of 571 patients living with HIV who experienced type 1 or type 2 MI (median age, 49 years [IQR, 43–55]; 430 men and 141 women), the distribution of MI types was nearly equal, with 50.4 % classified as type 2 MI and 49.6 % as type 1 MI [9]. Our findings from the Etiological Classification of identified MI were remarkably consistent with the above study. We observed that 50 % of all MIs were classified as type 1 MI and 45 % classified as type 2 MI.

Among the MIs identified using the Binary Classification in our cohort (*N* = 333), approximately half were reclassified as type 1 MI (*N* = 169) under the Etiological Classification system. The remaining events were classified as type 2 MI (*N* = 77), acute non-ischemic myocardial injury (*N* = 55), other injury types (*N* = 18), or no myocardial injury (*N* = 14). We observed that when adjudication is limited to the Binary Classification (presence or absence of MI), the majority of MI events were defined as type 1 MI. This is consistent with the work of McCarthy et al. [15], that reported significant differences in the frequency, characteristics, and outcomes of patients coded as acute MI in clinical practice prior to 2017 (MI type not specified) to the same ICD MI codes post 2017 (at which time these code was specified as type 1 MI and distinct codes for alternative MI types were available, e.g. type 2 MI). The findings of our study and those of McCarthy et al. are supportive of the hypothesis that classification of MI is dependent on the availability of other myocardial injury categories from which to choose from [[15], [16]].

In our analysis, the MIs (type 1–5) adjudicated by the Etiological Classification were classified as an MI (single entity) in the Binary Classification system in about two-thirds of cases. In MESA and other similar cardiovascular cohorts, the classification of MI types 1–5 with the Etiological Classification will allow investigation into how discoveries based on the binary definition “MI” translate to distinct MI types defined by their underlying etiology. Furthermore, less than half of the type 2 MIs identified by the Etiological Classification were captured as MIs in the Binary Classification. This highlights the value of an etiological classification system to capture and identify a larger, more complete group of etiologically distinct myocardial injuries. A few type 1 MIs (*N* = 33) diagnosed by the Etiological Classification were diagnosed as no MI in the Binary Classification. Only a very small number of events classified as MI by Binary Classification (*N* = 10) were classified as no myocardial injury by Etiological Classification. This may reflect an appreciation for “smaller” MIs at the time of adjudication via Etiological Classification versus Binary Classification, specific biomarker criteria differences between the two adjudication systems, or other differences between the adjudication panels performing case evaluations at a different time in medicine, 2000–2014 versus 2022–2025.

Similar to cardiovascular cohort studies, with rare exception [[17], [18]], clinical trials in acute MI have largely been limited to “acute MI” as a singular outcome, rather than etiologically specific MI types (e.g., type 1 or 2 MI). Prior cohort studies adjudicating MI as type 1 or type 2 have primarily been limited to evaluating patients at the time of an acute presentation, restricting associations to cross-sectional analysis and incident data limited to patients presenting with an acute event, rather than an assessment of the general adult population [[19], [20], [21]] Consequently, data on incidence and predisposing risk factors for etiology-specific types of MI or acute non-ischemic myocardial injury is limited [22,2].

In addition to the scientific and patient care implications of MI misclassification and the absence of fundamental epidemiological data for the full spectrum of myocardial injury events, MI classification also has operational implications for healthcare in the US. While effective, time-sensitive, evidence-based treatments exist for type 1 MIs, such data for type 2 MI and acute non-ischemic myocardial injury are limited. Consequently, patients with type 1 MI (but not type 2 MI or acute non-ischemic myocardial injury) are subject to clinical performance and quality measures, and value-based programs linking Medicare payment to the quality-of-care metrics. The application of these metrics requires an accurate classification of myocardial injury events. Currently, individual health systems are utilizing their own standards to make this distinction. Standardizing Etiological Classification-based adjudication across research and clinical practice may therefore improve comparability across studies, refine population health estimates, and ultimately inform evidence-based policy decisions aimed at improving care across the spectrum of myocardial injury events.

### 4.1 Strengths and limitations

This study has some limitations. A direct comparison of the two adjudication systems must be interpreted with caution, as they were designed with different goals, the Binary Classification focuses on identifying the presence of an MI, while the Etiological Classification categorizes all myocardial injury events based on the underlying etiology. The criteria for adjudicating MI and the personnel applying the criteria differed between the two classification systems. MESA began before the 99^th^ percentile URL was widely adopted as the standard for abnormal cardiac troponin. Adjudicators were limited to on-site-reported assay decision limits for cTn, which may differ from the manufacturer's 99^th^ percentile URL. The criteria for biomarker elevation and change differ between the Historical and Etiological Classification. Similar to the adjudication of all events occurring in research, the evaluation of cases is restricted to the data collected by the patient's non-study care team. However, in our study, the record collection for all events reviewed was thorough and included ECG or cardiac biomarkers for 100 % of cases classified as a myocardial injury [12]. While clinical reports for imaging data (e.g. angiograms, echocardiogram) were available for adjudicator review, the imaging themselves were not available.

## 5 Conclusions

Despite the prevalence and high mortality of multiple types of myocardial injuries, the identification and classification of acute myocardial injury events in community-based prospective cardiovascular cohorts are conspicuously lacking. The Etiological Classification system of myocardial injury evaluated here identifies a broader and more clinically meaningful spectrum of myocardial injury phenotypes than a Binary Classification system. Integration of Etiological Classification into prospective cohort studies and future clinical trials will allow for more etiology-based understanding of these distinct but similarly common group of cardiovascular events. Such data allow for specific acute myocardial injury type risk prediction, prevention, diagnosis, and treatment that simply cannot be achieved with Binary Classification.

## Funding/ support

This research was supported by grant 5R01HL158976–04 from the National Heart, Lung, and Blood Institute. MESA is supported by contracts 75N92025D00022, 75N92020D00001, HHSN268201500003I, N01-HC-95159, 75N92025D00026, 75N92020D00005, N01-HC-95160, 75N92020D00002, N01-HC-95161, 75N92025D00024, 75N92020D00003, N01-HC-95162, 75N92025D00027, 75N92020D00006, N01-HC-95163, 75N92025D00025, 75N92020D00004, N01-HC-95164, 75N92025D00028, 75N92020D00007, N01-HC-95165, N01-HC-95166, N01-HC-95167, N01-HC-95168 and N01-HC-95169 from the National Heart, Lung, and Blood Institute, and by grants UL1-TR-000040, UL1-TR-001079, and UL1-TR-001420 from the National Center for Advancing Translational Sciences (NCATS). The authors thank the other investigators, the staff, and the participants of the MESA study for their valuable contributions. A full list of participating MESA investigators and institutions can be found at http://www.mesa-nhlbi.org or https://biolincc.nhlbi.nih.gov.

## CRediT authorship contribution statement

**Shubham Tomar:** Writing – review & editing, Writing – original draft, Visualization, Methodology, Investigation, Formal analysis, Data curation, Conceptualization. **Karita C.F. Lidani:** Writing – review & editing, Visualization, Methodology, Data curation. **Matthew Dekker:** Writing – review & editing, Visualization, Data curation. **Aline A.I. Moraes:** Writing – review & editing, Methodology, Data curation. **Wendy S. Post:** Writing – review & editing, Data curation. **Patrick J. Trainor:** Writing – review & editing, Data curation. **Craig W. Johnson:** Writing – review & editing, Validation, Investigation, Data curation. **Susan R. Heckbert:** Writing – review & editing, Validation, Investigation, Data curation. **Roger S. Blumenthal:** Writing – review & editing, Validation, Investigation, Data curation. **Robyn L. McClelland:** Writing – review & editing, Validation, Investigation, Data curation. **Yunbi Nam:** Writing – review & editing, Data curation. **Khurram Nasir:** Writing – review & editing, Validation, Investigation, Data curation. **Michael J. Blaha:** Writing – review & editing, Supervision, Methodology, Investigation, Data curation. **Andrew P. DeFilippis:** Writing – review & editing, Validation, Supervision, Resources, Project administration, Methodology, Investigation, Funding acquisition, Data curation, Conceptualization.

## Declaration of competing interest

The authors declare the following financial interests/personal relationships which may be considered as potential competing interests: Andrew P. DeFilippis reports financial support was provided by National Heart Lung and Blood Institute. If there are other authors, they declare that they have no known competing financial interests or personal relationships that could have appeared to influence the work reported in this paper.

## Appendix Supplementary materials

Image, application 1
