# Supplementary material for: Etiological myocardial injury classification versus binary myocardial infarction classification in the multi-ethnic study of atherosclerosis (MESA)
Source: Am J Prev Cardiol. 2026 Mar 4;27:101525. doi: 10.1016/j.ajpc.2026.101525 (PMC13261280; doi:10.1016/j.ajpc.2026.101525)
Supplement: Supplementary file 1 [file mmc1.docx]

**Supplement Methods:**

Identification of events:

The cardiovascular outcomes of primary interest of Multi-Ethnic Study of Atherosclerosis (MESA) are myocardial infarction (MI), angina, congestive heart failure (CHF), peripheral vascular disease (PVD), stroke, transient ischemic attack (TIA), and cardiovascular-related deaths. Although other events—such as revascularization procedures, non-cardiovascular hospitalizations, and non-CVD deaths—are documented, they are not primary outcomes. The MESA identifies potential cardiovascular events (including CHF, angina, PVD, MI, stroke, TIA) through a combination of participant self-reports during in-person examinations or clinic visits, interim follow-up telephone interviews conducted every 9 to 12 months, direct notifications from participants to field centers following an event, and the identification of ICD-9 and ICD-10 codes associated with cardiovascular diseases by a computer algorithm. For participants who become lost to follow-up, the Field Center rarely monitors obituaries or public records to identify deaths. Once an event is suspected, MESA Surveillance and Events staff review and abstract detailed medical records, including physician notes, diagnostic test results (e.g., electrocardiograms and coronary angiography), discharge summaries, laboratory findings, and billing codes as per defined protocol. MESA’s event detection process is intentionally designed to prioritize sensitivity—even at the cost of specificity—to ensure that all potential cardiovascular events are flagged for review.

Adjudication of MI in Binary Classification:

All possible clinical events in MESA Binary Classification were independently reviewed by two physicians and categorized as “MI” (Definite or Probable MI) or “No/Absent MI”. Disagreements trigger a re-review by the final reviewer; if unresolved, a third reviewer adjudicates. Persistent disagreement is resolved by the MESA Morbidity and Mortality Committee. In MESA, cardiovascular event adjudication, including the Binary Classification of MI, has been performed till date since 1999 as part of an ongoing standardized protocol.

The MESA criteria for MI adjudication have been adapted from the Atherosclerosis Risk in Communities (ARIC) Study. The source for the ARIC criteria is: “ARIC Protocol 3, Surveillance Component Procedures, Version 4.0” (October 1997), which relies on: chest pain, cardiac biomarkers, and ECG findings.

Chest pain is considered present if it reflects typical ischemic symptoms (ischemic pain, tightness, pressure, or discomfort in the chest, arm, or jaw) or atypical symptoms deemed ischemic by clinicians, while clearly non-cardiac pain is excluded. Duration of pain is not considered part of the chest pain criteria.

Detailed cardiac biomarker criteria are in **Supplement Table 4**. The cardiac biomarker criteria in the Binary Classification did not require the alteration to be dynamic (rise or fall). Cardiac biomarkers (and cutoff range) measured during the time of event were used for adjudication. Broadly, cardiac biomarker abnormalities were categorized as equivocal if between the upper limit of normal (ULN) and twice the ULN, and as abnormal if greater than twice the ULN, with specific criteria for different enzymes as mentioned in supplement table 1. For procedures like PCI and CABG, separate thresholds apply: for PCI, CK or MB above three times the ULN within 48 hours is considered abnormal; for CABG, MB must exceed five times the ULN within 48 hours. These values are not downgraded due to the procedure. After 48 hours, the standard biomarker criteria would again apply.

ECGs are reviewed from multiple time-points, including the first two codable ECGs after admission, the last before discharge, and one from day 3 or later. Each adjudicator reviews ECGs independently.

A separate category exists for resuscitated cardiac arrest, which is assigned to patients who experienced full arrest (e.g., asystole or ventricular fibrillation), were successfully resuscitated, regain consciousness, and survive to hospital discharge. Arrests due to non-cardiac causes are excluded, and MI is generally not adjudicated in these cases due to the confounding effects of post-arrest biomarker elevations.

Participants who do not regain consciousness and subsequently die are classified based on cause of death, not as resuscitated cardiac arrest. Fatal MI and CHD events in MESA are classified based on the absence of a non-cardiac or non-atherosclerotic cause of death. A definite fatal MI includes in-hospital deaths meeting MI criteria or out-of-hospital deaths with a documented MI in the past 28 days. Definite fatal CHD requires recent chest pain or a history of CHD but does not meet criteria for fatal MI. Possible fatal CHD includes deaths without the above criteria but with CHD-related ICD-9 or 10 codes.

Per MESA protocol, when two inpatient hospitalizations occur within 30 days and have similar underlying conditions, the MESA Surveillance and Events staff flag them for combined adjudication—this process is referred to as “linking” two events. While MESA reviews recurrent events as if they are new, in rare cases where the hospitalizations are close to each other and clinically related (e.g., an admission for arterial occlusion followed shortly by a readmission for a revascularization procedure), field centers may combine them into a single event.

Adjudication of myocardial injury in the Etiological Classification:

Adjudication of myocardial injury through Etiological Classification required agreement between two cardiologists. In case of disagreement, a third cardiologist assessed the event; if their classification matched either of the previous two, consensus was reached. If all three reviewers differed, an expert advisory board consulted with the others to make the final determination. A separate adjudication team performed the Etiological Classification between 2022 and 2025, independently and blinded to the results of the Binary Classification.

The Etiological Classification of myocardial injury is based on the taxonomy outlined in the Fourth Universal Definition of Myocardial Infarction (4^th^ UDMI), which categorizes events into nine etiologically distinct subtypes.^1^ The adjudication approach, adapted from the method by Spatz et al. and detailed in *Circulation*, relied exclusively on clinical information collected by the MESA Coordinating Center (as described in “Identification of events” above).^2,3^ Data collected solely for research, including advanced diagnostics, such as genetic markers or novel imaging techniques, were not used in the adjudication process. Each event eligible for adjudication underwent a thorough independent review using all original documentation, including H&P notes, ECGs, and cardiac imaging reports.

To support the Etiological adjudication process, a web-based adjudication tool was developed in REDCap to standardize data collection and classification. The embedded algorithm ensures that each event meets minimum criteria for classification. Although an event may meet criteria for multiple categories, only one final adjudicated diagnosis is assigned. If a reviewer’s final decision does not align with the algorithm, the tool prompts a re-review and requires a written explanation to justify the classification.

A more through description of the study design and Etiological adjudication process has been previously published.

**Supplement Table 1: Specific types of myocardial injury in Etiological Classification.**

| Type 1 MI | MI caused by atherosclerotic plaque disruption (rupture or erosion) resulting in coronary thrombosis.  Features on coronary angiography that are consistent with coronary thrombosis include: spherical, ovoid, or irregular filling defect; abrupt vessel cutoff; intraluminal staining; and any coronary filling defect (PMID: 28150291). |
| --- | --- |
| Type 2 MI | MI caused by a mismatch between oxygen supply and demand by a pathophysiological mechanism other than coronary atherothrombosis (Type 1 MI) sustained and severe enough to cause ischemia (inadequate oxygen supply to the myocardium).  **Coronary causes**: spasm, embolism, dissection, microvascular disease.  **Cardiac (non-coronary):**   - significant tachy or brady arrhythmias sustained and relatively severe enough to be responsible for ischemia. - severe aortic stenosis/acute valve disease.   **Extra-cardiac:** anemia, thyroid storm, severe hypertension, hypoxemia, seizure resulting in hypoxemia and/or hypotension. |
| Type 3 MI | Cardiac death, with symptoms suggestive of myocardial ischemia accompanied by presumed new ischemic ECG changes or ventricular fibrillation, but death occurs before blood samples for biomarkers can be obtained, or before increases in cardiac biomarkers can be identified, or MI is detected by autopsy examination. |
| Type 4a MI | PCI related MI that is not stent thrombosis or restenosis. |
| Type 4b MI | Stent thrombosis. |
| Type 4c MI | Stent restenosis. |
| Type 5 MI | MI in setting of CABG not from other identifiable cause. |
| Acute Non-Ischemic  Myocardial Injury | **Direct and acute myocardial toxicity/insult**   - Acidosis. - Sepsis in the absence of type 2 MI from sustained profound hypotension or tachyarrhythmia. - Post-op state in the absence of type 2 MI from sustained profound hypotension or tachyarrhythmia. - Myocarditis/inflammation. - Heart failure in the absence of type 1 or type 2 MI. - Stress cardiomyopathy in the absence of a cause consistent with type 2 MI (coronary spasm). - Direct catecholamine toxicity: intracranial bleed, stress cardiomyopathy. - Cardiac contusion from trauma, ablation. |
| Chronic Myocardial Injury | - Renal failure - Heart failure - LVH, severe HTN - Infiltrative cardiomyopathy (amyloid, sarcoid, hemochromatosis) - Prostration/failure to thrive |

*CABG, Coronary Artery Bypass Grafting; ECG, Electrocardiogram; HTN, Hypertension; LVH, Left Ventricular Hypertrophy; MI, Myocardial Infarction; PCI, Percutaneous Coronary Intervention*

**Supplemental Table 2: Summary of clinical data availability and abstraction in the Etiological Classification.**

| *Characteristic* | Overall (N = 745) | Myocardial Injury (N = 669) | No Myocardial Injury (N = 76) |
| --- | --- | --- | --- |
| ***ECG available*** |  |  |  |
| *Image* | 689 (92.5%) | 620 (92.7%) | 69 (90.8%) |
| *Report only* | 47 (6.3%) | 43 (6.4%) | 4 (5.3%) |
| *None available* | 9 (1.2%) | 6 (0.9%) | 3 (3.9%) |
| ***STEMI status*** |  |  |  |
| *NSTEMI* | 631 (84.7%) | 558 (83.4%) | 73 (96.1%) |
| *STEMI* | 105 (14.1%) | 105 (15.7%) | 0 (0.0%) |
| *ECG not available* | 9 (1.2%) | 6 (0.9%) | 3 (3.9%) |
| ***Myocardial image available*** | 538 (72.2%) | 496 (74.1%) | 42 (55.3%) |
| *ECHO* | 474 (63.6%) | 445 (66.5%) | 29 (38.2%) |
| *VGRAM* | 124 (16.6%) | 113 (16.9%) | 11 (14.5%) |
| *NUC* | 17 (2.3%) | 15 (2.2%) | 2 (2.6%) |
| *MRI* | 1 (0.1%) | 1 (0.1%) | 0 (0.0%) |
| ***Stress test available*** | 79 (10.6%) | 70 (10.5%) | 9 (11.8%) |
| ***Coronary Angiogram available*** | 306 (41.1%) | 276 (41.3%) | 30 (39.5%) |
| ***Imaging (****ECHO or VGRAM or NUC or MRI or Stress test or CATH****)*** | 538 (72.2%) | 496 (74.1%) | 42 (55.3%) |
| ***Cardiac Biomarker*** *(Total CK or CKMB or Troponin-T or Troponin-I)* | 698 (93.7%) | 636 (95.1%) | 62 (81.6%) |
| *Total CK* | 473 (63.5%) | 427 (63.8%) | 46 (60.5%) |
| *CK-MB* | 431 (57.9%) | 389 (58.1%) | 42 (55.3%) |
| *cTn (Troponin T or Troponin I)* | 688 (92.3%) | 630 (94.2%) | 58 (76.3%) |
| *Total CK or CKMB* | 518 (69.5%) | 469 (70.1%) | 49 (64.5%) |
| *Total CK or cTn* | 696 (93.4%) | 635 (94.9%) | 61 (80.3%) |
| *CKMB or cTn* | 696 (93.4%) | 634 (94.8%) | 62 (81.6%) |
| ***Combinations*** |  |  |  |
| *ECG or Imaging or Cardiac Biomarker* | 745 (100.0%) | 669 (100.0%) | 76 (100.0%) |
| *ECG or Cardiac Biomarker* | 745 (100.0%) | 669 (100.0%) | 76 (100.0%) |
| *ECG or Imaging* | 723 (97.0%) | 657 (98.2%) | 66 (86.8%) |
| *Imaging or Biomarker* | 513 (68.9%) | 475 (71.0%) | 38 (50.0%) |
| *ECG & Imaging & Biomarker* | 698 (93.7%) | 636 (95.1%) | 62 (81.6%) |
| *ECG & Biomarker* | 538 (72.2%) | 496 (74.1%) | 42 (55.3%) |
| *ECG & Imaging* | 513 (68.9%) | 475 (71.0%) | 38 (50.0%) |
| *Imaging & Biomarker* | 538 (72.2%) | 496 (74.1%) | 42 (55.3%) |

*CATH, Cardiac Catheterization; cTn, Cardiac Troponin; CK-MB, Creatinine Kinase-Myocardial Band; ECHO, Echocardiogram; ECG, Electrocardiogram; MRI, Magnetic Resonance Imaging; NSTEMI, Non-ST-segment Elevation Myocardial Infarction; NUC, Nuclear Scan; STEMI, ST-segment Elevation Myocardial Infarction; Total CK, Total Creatinine Kinase; VGRAM, Ventriculogram*

*Note: As per design, no clinical covariates were collected on ‘no myocardial injury’ events which were concordant amongst initial two adjudicators in Etiological Classification (N= 3,330)*

**Supplemental Table 3: Summary of clinical data availability and abstraction in the Etiological Classification for Type 1 MI, Type 2 MI, and Acute non-ischemic myocardial injury.**

| ***Characteristic*** | ***Overall (N = 582)*** | ***Type 1 MI (N = 202)*** | ***Type 2 MI (N = 182)*** | ***Acute non-ischemic myocardial injury (N = 198)*** |
| --- | --- | --- | --- | --- |
| ***ECG available*** |  |  |  |  |
| *Image* | *540 (93%)* | *189 (94%)* | *171 (94%)* | *180 (91%)* |
| *Report only* | *38 (6.5%)* | *13 (6.4%)* | *9 (4.9%)* | *16 (8.1%)* |
| *None available* | *4 (0.7%)* | *0 (0%)* | *2 (1.1%)* | *2 (1.0%)* |
| ***Imaging (ECHO or VGRAM or NUC or MRI or Stress test or CATH)*** | *448 (77%)* | *183 (91%)* | *133 (73%)* | *132 (67%)* |
| ***Myocardial image available*** | *448 (77%)* | *183 (91%)* | *133 (73%)* | *132 (67%)* |
| *ECHO* | *399 (69%)* | *142 (70%)* | *131 (72%)* | *126 (64%)* |
| *VGRAM* | *105 (18%)* | *83 (41%)* | *6 (3.3%)* | *16 (8.1%)* |
| *NUC* | *14 (2.4%)* | *2 (1.0%)* | *4 (2.2%)* | *8 (4.0%)* |
| *MRI* | *1 (0.2%)* | *1 (0.5%)* | *0 (0%)* | *0 (0%)* |
| ***Stress test available*** | *66 (14%)* | *22 (12%)* | *23 (17%)* | *21 (15%)* |
| ***Coronary Angiogram available*** | *247 (42.4%)* | *173 (85.6%)* | *33 (18.1%)* | *41 (20.7%)* |

*Imaging: Myocardial imaging (ECHO or VGRAM or NUC or MRI) or stress test or CATH; CATH, Cardiac Catheterization; ECHO, Echocardiogram; ECG, Electrocardiogram; MI, Myocardial Infarction; MRI, Magnetic Resonance Imaging; NSTEMI, Non-ST-segment Elevation Myocardial Infarction; NUC, Nuclear Scan; STEMI, ST-segment Elevation Myocardial Infarction; VGRAM, Ventriculogram*

**Supplement Table 4:** MESA Algorithm to Classify Cardiac Enzymes as Abnormal, Equivocal, or Normal.

| **Enzyme Value** | **There is (a) no known muscle trauma/ hemolysis and (b) no PTCA or CABG in past 48 hours*** | **Muscle trauma/ liver/ hemolytic disease exists** |
| --- | --- | --- |
| CK-MB = present where present or absent | Abnormal | Equivocal |
| CK-MB ≥ 2× ULN | Abnormal | Equivocal |
| CK-MB** ≥ 10% Total CK, if no ULN is given | Abnormal | Equivocal |
| Total CK ≥ 2× ULN and LDH ≥ 2× ULN | Abnormal | Equivocal |
| LDH-1: LDH-2 > 1 | Abnormal | Equivocal |
| LDH-1 ≥ 2× ULN if LDH-2 is missing | Abnormal | Equivocal |
| Total CK ≥ 2× ULN or LDH ≥ 2× ULN | Equivocal | Normal |
| Normal < Total CK < 2× ULN and Normal < LDH < 2× ULN | Equivocal | Normal |
| 5% Total CK < CK-MB^†^ < 9% Total CK or CK-MB “weakly present” | Equivocal | Equivocal |
| Normal < CK-MB < 2× ULN | Equivocal | Equivocal |
| Normal < LDH 1 < 2× ULN | Equivocal | Equivocal |
| Data present, but insufficient for above criteria | Incomplete | Incomplete |
| Troponins > 2× ULN | Abnormal | Abnormal |
| Troponins < ULN | Normal | Normal |
| CK-MB < ULN | Normal | Normal |
| All other results | Normal | Normal |

**PTCA–abnormal in first 48 hours requires Troponins or LDH-1 or CK or CK-MB > 3× ULN; equivocal requires 1-3× ULN.*

*CABG–abnormal in first 48 hours requires Troponins or LDH-1 or CK-MB > 5× ULN; equivocal requires 1-5× ULN*

*^†^CK and CK-MB must be in same units for this criterion.*

*CABG. Coronary Artery Bypass Grafting; CKMB, Creatine Kinase-MB Isoenzyme; LDH, Lactate Dehydrogenase; PTCA, Percutaneous Transluminal Coronary Angioplasty; Total CK, Total Creatine Kinase; ULN, Upper Limit of Normal*

**Supplement Table 5:** MESA Diagnostic Criteria for Hospitalized MI.

1. **Cardiac Pain Present**

| ***ECG Pattern*** | **Cardiac Enzymes** | | | |
| --- | --- | --- | --- | --- |
|  | **Abnormal** | **Equivocal** | **Incomplete** | **Normal** |
| Evolution of Major Q-Wave | Definite MI | Definite MI | Definite MI | Definite MI |
| Evolution of ST-segment Elevation with or without Q-wave  or  New LBBB | Definite MI | Probable MI | Probable MI | No MI |
| Evolution of ST-segment T-wave Depression/inversion alone  or  Evolution of Minor Q-waves alone | Definite MI | Probable MI | No MI | No MI |
| Single ECG with Major Q-Wave  or  Single ECG with LBBB, described as new | Definite MI | Probable MI | No MI | No MI |
| Normal, Absent, Uncodable, other | Probable MI | No MI | No MI | No MI |

1. **Cardiac Pain Absent**

| ***ECG Pattern*** | **Cardiac Enzymes** | | | |
| --- | --- | --- | --- | --- |
|  | **Abnormal** | **Equivocal** | **Incomplete or missing** | **Normal** |
| Evolution of Major Q-Wave | Definite MI | Definite MI | Definite MI | Definite MI |
| Evolution of ST Elevation with or without Q-wave  or  New LBBB | Definite MI | Probable MI | No MI | No MI |
| Evolution of ST-T Depression/inversion alone  or Evolution of Minor Q-Wave alone | Probable MI | No MI | No MI | No MI |
| Single ECG with Major Q-Wave  or  Single ECG with LBBB, described as new | Probable MI | No MI | No MI | No MI |
| Normal, Absent, Uncodable, other | Probable MI | No MI | No MI | No MI |

*LBBB, Left Bundle Branch Block; ECG, Electrocardiogram*

**Supplemental Table 6: Original adjudicated diagnosis in Etiological Classification of second event in the bundled event pairs that occurred within 28 days of discharge from previous myocardial injury event.**

| Etiological Classification diagnosis prior to bundling* | N (%) |
| --- | --- |
| Type 1 myocardial infarction | 5 (27.8) |
| Type 2 myocardial infarction | 2 (11.1) |
| Acute non-ischemic myocardial injury | 5 (27.8) |
| Chronic myocardial injury | 6 (33.3) |
| Total | 18 (100) |

**irrespective of the myocardial injury event that occurred within the prior 28 days*


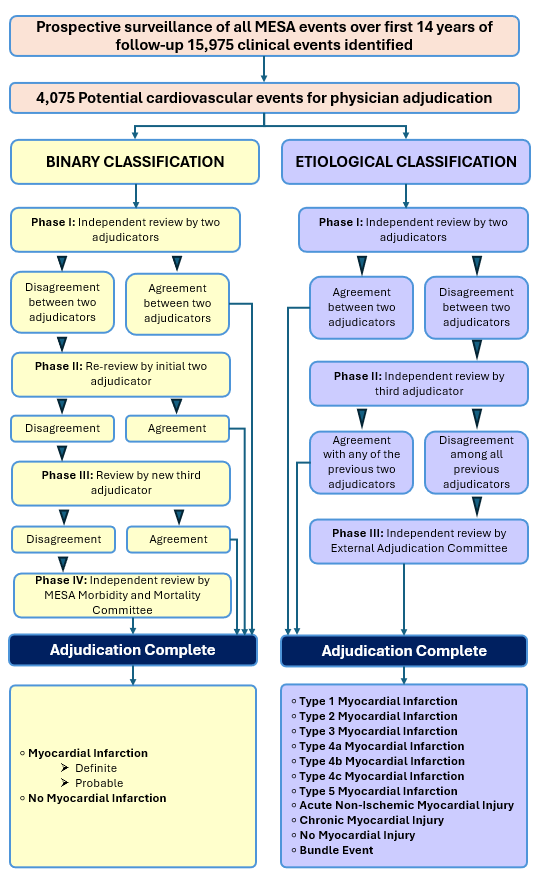


**Supplement Figure S1**: Event selection for physician adjudication and adjudication methodology in the Multi-Ethnic Study of Atherosclerosis. (Note: Bundling of events was done after adjudication was completed for all events. Adjudicators reviewed events occurring within 28 days of each other to determine if they are related or independent of each other)


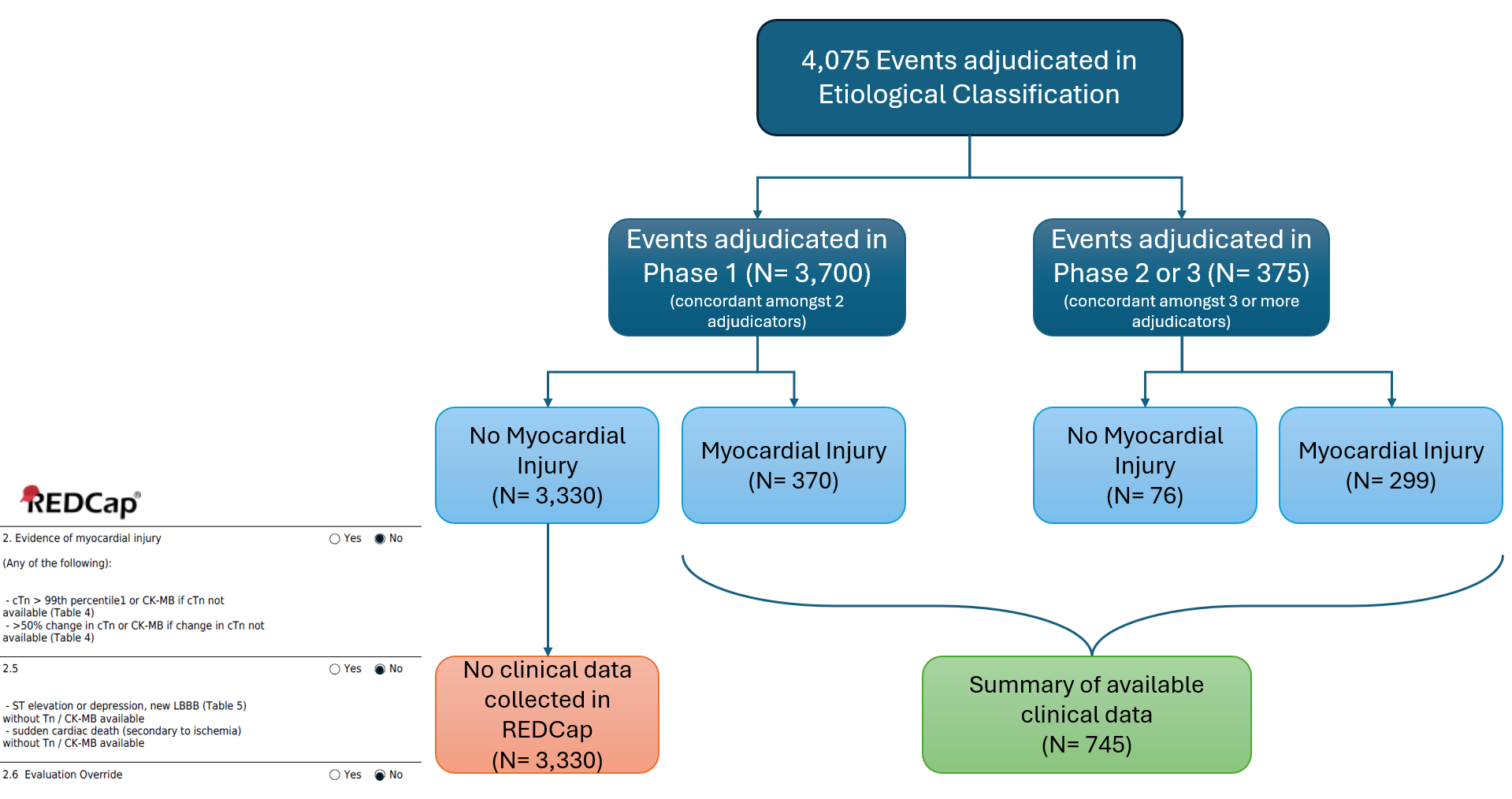


**Supplement Figure S2**: Flowchart of events adjudicated in Etiological Classification and clinical data availability
